# Protocol for a multi-country retrospective observational paediatric sepsis epidemiological study (SENTINEL International)

**DOI:** 10.1136/bmjopen-2025-101332

**Published:** 2025-10-15

**Authors:** Elliot Long, Amanda Williams, Shane George, Stephen Hearps, Adriana Yock-Corrales, Viviana Pavlicich, Kandamaran Krishnamurthy, Yashica Seymour-Hanna, Radhika Raman, Bharat Choudhary, Weda Kusuma, Victoria Ribaya, Nilanka Mudithakumara, Nichkamol Lertamornkitti, Antionette David, Suleiman Mohamed, Tigist Bacha Heye, Jenala Njirammadzi-Maleta, John Adabie Appiah, Janvier Hitayezu, Mukami Kunga, Genevieve Fung, Rahimi Abdrazak, Shu-Ling Chong, Jhong-Lin Wu, Jeong-Yong Lee, Damian Roland, Silvia Bressan, Ruth Loellgen, Yolanda Ballestero, Ozlem Teksam, Lina Jankauskaite, Graham Thompson, Vikram Sabhaney, Yasaman Shayan, Elysha Pifko, Julia Lloyd, Khalid Al Ansari, Franz E Babl

**Affiliations:** 1 Emergency, The Royal Children’s Hospital Melbourne, Melbourne, Victoria, Australia; 2 Clinical Sciences, Murdoch Children’s Research Institute, Parkville, Victoria, Australia; 3 Paediatrics, The University of Melbourne, Melbourne, Victoria, Australia; 4 Critical Care, The University of Melbourne, Melbourne, Victoria, Australia; 5 GCUH, Southport, Queensland, Australia; 6 Griffith University Menzies Health Institute Queensland, Gold Coast, Queensland, Australia; 7 Child Health Research Centre, The University of Queensland, Brisbane, Queensland, Australia; 8 Hospital Nacional de Niños, San Jose, Costa Rica; 9 Emergency Department, Universidad del Pacífico, Asunción, Paraguay; 10 PICU, The University of the West Indies, Bridgetown, St michael, Barbados; 11 Princess Margaret Hospital, St Michael, Bahamas; 12 Kanchi Kamakoti Childs Trust Hospital, Chennai, India; 13 AIIMS Jodhpur, Jodhpur, RJ, India; 14 Departemen Ilmu Kesehatan Mata RSUP DR Sardjito, Yogyakarta, Jogja, Indonesia; 15 Emergency, Philippine Children's Medical Center, Quezon City, Metro Manila, Philippines; 16 Lady Ridgeway Hospital for Children, Colombo, WP, Sri Lanka; 17 Department of Pediatrics, Faculty of Medicine, Siriraj Hospital, Mahidol Universitycity, Bangkok, Bangkok, Thailand; 18 Emergency, Colonial War Memorial Hospital, Suva, Central Division, Fiji; 19 Muhimbili National Hospital, Dar es Salaam, Dar es Salaam, Tanzania, United Republic of; 20 Pediatrics, Addis Ababa University, Addis Ababa, Oromia, Ethiopia; 21 Paediatric Intensive Care, Kamuzu University of Health Sciences, Blantyre, Southern Region, Malawi; 22 Paediatric Intensive Care, Komfo Anokye Teaching Hospital, Kumasi, Ashanti Region, Ghana; 23 Emergency, CHUK, Kigali, Kigali City, Rwanda; 24 Paediatric Critical Care & Emergency Department, Kenyatta National Hospital, Nairobi, Nairobi County, Kenya; 25 Paediatric Emergency, Prince of Wales Hospital Department of Medicine and Therapeutics, Hong Kong, Hong Kong; 26 Hospital Tuanku Ja’afar Seremban, Seremban, Negeri Sembilan, Malaysia; 27 Department of Emergency Medicine, KK Women's and Children’s Hospital, Singapore; 28 Emergency, National Taiwan University Hospital, Taipei City, Taipei City, Taiwan; 29 Emergency, Asan Medical Center Children’s Hospital, Songpa-gu, Seoul, Korea (the Republic of); 30 Emergency Department, Paediatric Emergency Medicine Leicester Academic (PEMLA) Group, Leicester, UK; 31 SAPPHIRE group, University of Leicester Department of Health Sciences, Leicester, UK; 32 Padua University Hospital, Padova, Italy; 33 Universita degli Studi di Padova Dipartimento di Salute della Donna e del Bambino, Padova, Italy; 34 Emergency, Astrid Lindgren Children’s Hospital, Stockholm, Stockholm County, Sweden; 35 Department of Women’s and Children’s health, Karolinska Institute, Stockholm, Stockholm County, Sweden; 36 Pediatric Emergency Department, Cruces University Hospital Paediatric Emergencies, Barakaldo, PV, Spain; 37 Emergency, Hacettepe İhsan Doğramacı Children’s Hospital, Ankara, Turkey; 38 Lithuanian University of Health Sciences Hospital Kauno klinikos, Kaunas, Kaunas County, Lithuania; 39 Pediatric Emergency Medicine, University of Calgary, Calgary, Alberta, Canada; 40 Emergency, British Columbia Children’s Hospital, Vancouver, British Columbia, Canada; 41 CHU Sainte-Justine, Montreal, Quebec, Canada; 42 Emergency, Nemours Children’s Hospital Delaware, Wilmington, Delaware, USA; 43 Department of Pediatrics, Nationwide Children’s Hospital, Columbus, Ohio, USA; 44 Sidra Medical and Research Center, Doha, Qatar

**Keywords:** Sepsis, Child, Epidemiology

## Abstract

**Introduction:**

Improving outcomes from sepsis in children is a WHO Global Health Priority, yet mortality from sepsis remains high, particularly in low- and middle-income countries (LMICs). This database from children with community-acquired childhood sepsis in LMICs and some high-income countries will allow analysis of the burden of disease, including incidence, severity and outcomes. Understanding these aspects of sepsis care is fundamental for the design and conduct of future international interventional trials to improve childhood sepsis outcomes.

**Methods and analysis:**

This multicountry retrospective observational study will include children up to 18 years of age presenting to emergency departments with suspected sepsis, defined as admission to hospital for treatment with intravenous antibiotics plus (1) a provisional diagnosis of sepsis and/or (2) treatment for suspected sepsis (operationalised as the administration of one or more fluid bolus to treat impaired perfusion or vasoactive infusion). Presenting characteristics, management and outcomes will be collected. These will include vital signs, serum biomarkers, intravenous fluid administration for the first 24 hours of hospitalisation, organ support therapies delivered, antimicrobial use, microbiological diagnoses, hospital and intensive care unit length of stay, and mortality censored at hospital discharge or 30 days from enrolment (whichever occurs first).

**Ethics and dissemination:**

Central ethics approval was received from the Royal Children’s Hospital of Melbourne, Australia Human Research Ethics Committee (HREC/100648/RCHM-2023). Each international site will be required to obtain local Institutional Research Ethics Board approval. The findings will be disseminated in peer-reviewed journals, at academic conferences and through lay media. A cleaned study database and individual site-level data will be made available to site investigators upon completion of the study.

**Trial registration number:**

This study was registered with the Australian and New Zealand Clinical Trials Registry on 23 January 2024 prior to commencement of recruitment (ACTRN12624000052538).

**STRENGTHS AND LIMITATIONS OF THIS STUDY**

- This study is being conducted across multiple emergency departments (EDs) primarily in low- and middle-income countries using standardised inclusion criteria, allowing comparison between populations.
- This study will demonstrate the feasibility of manual data extraction and transfer from multiple countries with different healthcare systems and research infrastructure.
- This study uses similar inclusion criteria to those that may be used in future interventional trials, paving the way for ongoing international collaborative research.
- The potential for variability in case identification and data collection has been mitigated using data dictionaries and standard operating procedures, site training and piloting of clinical report forms, delegation logs and ongoing data quality monitoring in keeping with good clinical practice.
- While multiple EDs are included, most are tertiary referral centres and may not reflect the population seen in community, rural and regional centres.

## Introduction

 Sepsis is an important global health issue. Over 25.2 million children per year worldwide develop sepsis, resulting in 3.4 million deaths, most of them in children younger than 5 years of age.^1^ The hospitalisation cost per child with sepsis in the USA alone is estimated at US$26 592, resulting in an annual expenditure of US$7.31 billion representing 18.1% of nationwide paediatric hospitalisation costs in the USA in 2019.^2^ Reducing childhood deaths from sepsis is a WHO Global Health Priority and is essential if the United Nations Sustainable Development Goals are to be achieved.^3^

Despite advances in public health, prevention and treatment of many infectious diseases, invasive infections and sepsis remain leading causes of preventable childhood death worldwide.^4^ Mortality from sepsis remains substantial, even in high-income countries (HICs).^5^ Children with sepsis are disproportionately affected in terms of disability-adjusted life years compared with adults with sepsis, due to premature death (years of life lost) and long-term disabilities in sepsis survivors (years lived with disability).^6^ Despite decades of research into the treatment of sepsis, sepsis-specific mortality remains unchanged. Any reduction in sepsis mortality mirrors that of children hospitalised for non-infectious causes.^7^ This may in be part due to an increasing number of patients surviving with high-risk conditions, such as extreme prematurity, acquired or innate immunodeficiency, or the presence of indwelling vascular catheters.^8^

Many challenges contribute to the lack of accurate reporting of sepsis epidemiology, severity and therapies in children, with the major contributor being inconsistency in the methods used for case ascertainment. International Classification of Disease codes have been used to retrospectively identify children with sepsis or infection-related organ dysfunction, with a sevenfold difference in incidence found between these two case ascertainment methods.^9^ Case ascertainment using International Sepsis Consensus Conference criteria has been found to be discordant with clinician ascertainment of sepsis, with an inter-rater agreement (kappa) of 0.57.^10^ Recently, the Society of Critical Care Medicine has derived and validated the international consensus-based Phoenix criteria to diagnose and report sepsis in children, based on measures of dysfunction of four organs after the initial 24 hours of hospitalisation.^11^ There remain questions, however, regarding the applicability of these criteria in the emergency department (ED) and early in hospitalisation, the impact of missing organ dysfunction data (particularly in low- and middle-income countries (LMICs)) and the test characteristics of the Phoenix sepsis criteria for predicting mortality.^12 13^ Ideally, data used as diagnostic criteria from across countries are collected in a way that allows comparison between populations.

In an international collaboration between emergency physicians, paediatricians and intensivists, this study will develop the first database for children with community-acquired childhood sepsis in LMIC and select HIC. We will describe sepsis prevalence, severity and outcomes. We will report current management practice and antimicrobial use. We will apply diagnostic criteria and risk stratification criteria to allow standardised comparisons across diverse populations. This study will provide the critical understanding of the epidemiology of children with suspected sepsis as a basis for future interventional trials.

## Methods

### Design

This is a multicountry retrospective observational study. Studies conducted using this database will follow the Strengthening the Reporting of Observational Studies in Epidemiology guidelines for the reporting of observational studies and the Standards for the Reporting of Diagnostic Accuracy studies (STARD) guidelines for the validation of sepsis organ dysfunction criteria using methodology applied to similar bi-national (Australia and New Zealand) sepsis database.^12 14 15^

### Patient and public involvement

The research team includes a parent consumer lead with lived experience caring for a child with infection-related conditions including sepsis. This parent consumer and a hospital family advisory committee reviewed the study for acceptability of data collection. Their feedback will be included in publications and presentations of study results. Study data and outcomes collected include those deemed of high importance to parents and clinicians.^16 17^

### Setting and participants

The study will take place at multiple EDs who individually see at least 20 000 children per year. Out of a total of 34 sites, 25 are in LMICs and 9 in HICs (see online supplemental Material). Study sites were identified through known contacts. No approached sites declined participation. The central coordinating site for the study is the Murdoch Children’s Research Institute, which is affiliated with The Royal Children’s Hospital Melbourne.

#### Inclusion and exclusion criteria

Patients up to 18 years of age who present to a participating ED for treatment of suspected sepsis will be included. Suspected sepsis will be defined as children with the intention to admit to hospital for parenteral antibiotics and (1) a provisional (admission) diagnosis of sepsis, septicaemia or septic shock AND/OR (2) treatment for sepsis, operationalised as treatment with one or more fluid boluses to treat impaired perfusion, not dehydration (defined as a fixed volume of fluid administered over <30 min) or vasoactive infusion. We chose these inclusion criteria because prior studies had indicated that an admission diagnosis of sepsis alone did not identify a substantial proportion of febrile children started on a vasoactive infusion and treated in the intensive care unit (ICU).^18^ We included ‘treatment for suspected sepsis’ as an inclusion criterion as prior studies had identified that a substantial proportion of patients with infection requiring ICU-level care did not have an admission diagnosis of sepsis.^18^ This was operationalised using the administration of a fluid bolus to treat poor perfusion as this was the initial treatment for sepsis recommended in all guidelines and pathways at participating sites in addition to the use of intravenous antibiotics.^19^ The requirement for the fluid bolus to treat impaired perfusion was included as prior studies identified dehydration as the most common indication for fluid bolus administration.^20^ These criteria were used in a bi-national sepsis epidemiological study (SENTINEL; Australia and New Zealand) and differ from the inclusion criteria used in the derivation and validation of the Phoenix sepsis criteria (suspected infection operationalised as receipt of parenteral antimicrobials and microbiological testing within the first 24 hours of encounter).

Patients who are not admitted through the ED (such as direct inter-hospital ICU transfers) and patients who are admitted to another hospital ward >24 hours prior to ED transfer will be excluded due to difficulty obtaining initial vital signs and biomarkers. Patients presenting with trauma will be excluded.

### Aims

Primary and secondary aims are listed in Box 1.

Box 1 **Primary and secondary aims**

**Primary aim**

To create an international sepsis database and collect data over a 1-year period

**Secondary aims**

To aggregate and analyse sepsis data and describe:

1. Sepsis prevalence, severity and outcomes in children hospitalised with suspected community-acquired sepsis.
2. The microbiology, antibiotic use patterns and antimicrobial stewardship practices in hospitalised children with suspected sepsis.
3. The management of hospitalised children with suspected sepsis.

To apply standardised diagnostic criteria to this international cohort of children with suspected sepsis (SIRS, Phoenix sepsis criteria)

To apply standardised risk stratification tools to this international cohort of children with suspected sepsis (SIRS, pSOFA, qSOFA, qPELOD-2, Phoenix sepsis score)

To develop feasible inclusion criteria and outcomes, and establish feasibility of data collection and transfer, to be used in the design and conduct of future interventional trials in children with suspected sepsis

pSOFA, paediatric Sequential Organ Failure Assessment; qPELOD-2, quick Paediatric Logistic Organ Dysfunction-2; qSOFA, quick Sequential Organ Failure Assessment; SIRS, systemic inflammatory response syndrome.

### Patient recruitment, study procedures and data collection

**Figure 1:**
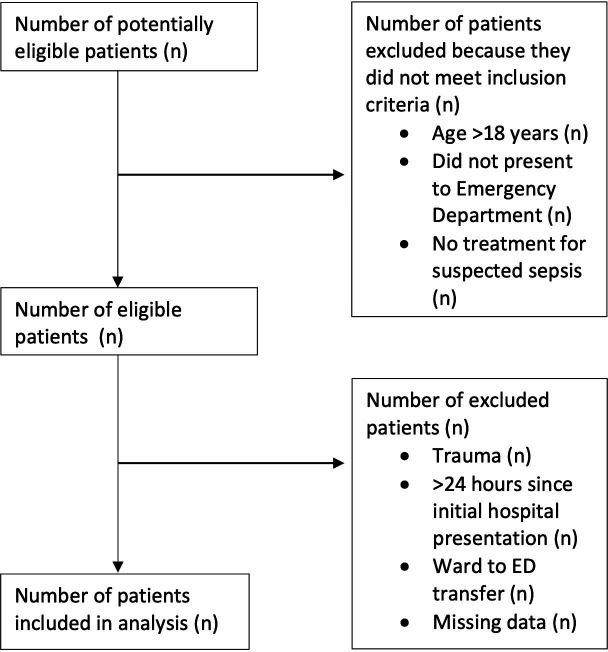
Participant enrolment flow chart. ED, emergency department.

ED attendance records will be screened at regular intervals, with a minimum frequency of every 14 days, by research staff to identify eligible patients (figure 1). The reference to missing data in the figure refers to the reason for exclusion. Participants meeting eligibility criteria will be enrolled in the study. Participant information will be stored in a secure, password-protected file at each local site, with personally identifiable information replaced with a unique study identification number.

Enrolled participants will have their medical records reviewed, and relevant de-identified data will be extracted and entered directly into a password-protected Research Electronic Data Capture (REDCap) electronic database. Data to be extracted are outlined in box 2. Age was measured in months for children <2 years of age and in years for children ≥2 years of age. Indigenous status was defined using local ethnicity criteria, where applicable (eg, Aboriginal or Torres Strait Islander in Australia; Māori or Pasifika in New Zealand). Comorbidities were defined using the Pediatric Complex Chronic Condition system.^21^ We included primary site of infection and primary pathogen causing sepsis in the data collected. We did not collect inflammatory markers such as C-reactive protein or procalcitonin.

Box 2 **Data to be collected by all participating EDs by time point**

**Initial ED attendance**

- Detailed patient demographic information (age, sex, Indigenous status, comorbidities)
- Vital signs (^β^HR, MBP, SBP, RR, SpO2, GCS score, CRT)
- Pathology tests (^χ^VBG, FBC, UEC, LFT, coagulation profile, troponin)
- Therapies administered (antibiotics, oxygen, fluids—bolus/maintenance/drug administration line, steroids, organ support)
- Admission diagnosis

**During hospital stay**

- Disposition (hospital ward, ICU)
- Vital signs (6, 12, 18 and 24 hours from ED arrival)
- Pathology tests (6, 12, 18 and 24 hours from ED arrival)
- Therapies administered (duration of hospitalisation censored at 30 days)
- ICU and hospital length of stay (censored at 30 days)
- Discharge diagnosis
- Results of all microbiological tests
- In-hospital mortality (censored at 30 days)

CRT, capillary refill time; ED, emergency department; FBC, full blood count; ICU, intensive care unit; LFT, liver function test; MBP, mean blood pressure; RR, respiratory rate; SBP, systolic blood pressure; UEC, urea electrolytes creatinine; ^α^GCS, Glasgow Coma Scale; ^β^HR, heart rate; ^χ^VBG, venous blood gas.

All research team members across participating sites will receive formal training by the central coordinating team, covering study processes and the completion of the REDCap database electronic clinical report form prior to commencing enrolment. Standardised teaching materials and procedures have been developed and will be distributed to all participating sites. A source data plan will be established at each site to ensure consistency in data. The central coordinating team will conduct monthly data audits, and any data queries will be communicated to the respective sites for resolution.

### Data management

Data collected from the medical records will be entered into a password-protected database enabled through the REDCap web-based application hosted by the Murdoch Children’s Research Institute (MCRI).^22^ This database will only be accessible to trained research staff. All data entered into this database will be de-identified. All sites will maintain a separate password-protected logbook on a local secure online database containing identifying information for data queries. Each site will have access to their individual site data only. Oversight of recruitment, study processes and compliance with data collection and data entry procedures will be conducted remotely, as detailed in the study manual of operations. If there is a need to re-identify data for clarification, this will be done by the principal investigator (PI) at the site level. All local data will be retained in line with the ethics and governance requirements of the local site. The Trial Steering Committee consisting of the Chief PI, trial coordinator and trial statistician will meet quarterly to discuss the progress of the study and review recruitment and data management.

## Statistical methods

### Sample size and power calculation

We aim to enrol more than 5000 children over 12 months. This will provide a large sample for describing the international sepsis landscape. No formal sample size calculation was undertaken due to lack of prior data regarding sepsis incidence. Where applicable, appropriate power calculations will be included in future papers describing specific outcomes using the database.

Descriptive statistics will be calculated for key epidemiological variables, using means and SD for normally distributed data, and medians and IQRs for skewed data. We will apply existing organ dysfunction criteria following the STARD and clinical decision rule guidelines.^15 23^ Organ dysfunction criteria will be based on the organs and cut-off points used in paediatric Sequential Organ Failure Assessment, Pediatric Logistic Organ Dysfunction-2(PELOD-2), quick Sequential Organ Failure Assessment, quick Paediatric Logistic Organ Dysfunction-2 and the Phoenix sepsis criteria.^24^ Completely missing values will be considered non-additive to organ dysfunction scores in keeping with their original validation studies.^11 25^ The level of significance will be set at p<0.05.

The feasibility of inclusion criteria and outcomes for use in the design and conduct of future international interventional trials will be assessed using prevalence, severity, outcome and treatment data from this study.

### Ethical issues, consent and dissemination

Approval for the conduct of this study was provided by the Royal Children’s Hospital Human Research Ethics Committee (HREC 100648) and is prospectively registered through the Australian and New Zealand Clinical Trials Registry (trial ID: ACTRN12624000052538). The MCRI serves as the primary sponsor for this trial. As this study is an international study, each participating site will be required to obtain local Institutional Research Ethics Board (IREB) approval. Each jurisdiction was consulted to determine the level of consent required. Given the observational non-interventional nature of this study, to date, no sites with IREB approval have been required to obtain consent. The study protocol will follow successful processes used for other large multicentre observational studies performed by the Paediatric Research in Emergency Departments International Collaborative network.^26^

### Risk management, adverse events and patient safety

As an observational study, there are no anticipated adverse events related to the research, except a minor risk of loss of confidentiality.

### Time plan

The study will collect data over a 1-year period (calendar year 2025) at all sites.

## Discussion

This is the first international database that will allow a comprehensive description of sepsis epidemiology. This will improve the understanding of global sepsis incidence, severity and outcomes, and allow for comparison between diverse populations. Prior global epidemiological studies have focused on the point prevalence of sepsis in critical care populations or global estimates of disease burden.^5 27^ Building on these prior efforts, we will explore differences in aetiology of sepsis and allow comparisons of management of sepsis including patterns of antimicrobial use between populations. Standardised criteria for sepsis diagnosis and risk stratification will be applied to the study cohort, providing a benchmark for between-population comparison. Finally, this study will provide baseline data and feasibility data for the conduct of future interventional trials.

This study has several limitations. Standard practice will be decided by the treating clinician, with inherent differences in thresholds for diagnosing and treating sepsis and therapies administered. Where no test for organ dysfunction was obtained at baseline in the ED, the result will be assumed to be normal, although this may not be the case. While multiple EDs are included in this study, most are tertiary referral centres and may not reflect the population seen in rural, community and regional centres. The study focuses on community-acquired sepsis, while hospital-acquired sepsis is a significant contributor to sepsis morbidity and mortality. Though we include participants <18 years, study sites may have different age thresholds for treating older adolescents, which may skew study age demographics. The study inclusion criteria differ from those used in the derivation and validation of the Phoenix sepsis criteria, which retrospectively collected data from the pre-COVID-19 era.^11^ We did not use microbiological testing as an inclusion criterion because many hospitals post COVID-19 performed routine microbiological testing in children in whom sepsis was not suspected. Nevertheless, our inclusion criteria when applied to two HICs identified remarkably similar cohort of patients to the Phoenix derivation and validation cohort.^12^ We did not collect data on inflammatory markers such as C-reactive protein or procalcitonin as they are not included in any sepsis diagnostic criteria or risk stratification criteria. We did not collect data including the Pediatric Index of Mortality-3 or Pediatric Risk of Mortality-3 as these scores were developed for use in the ICU setting.^28 29^ We did not collect any socioeconomic status measurements, limiting the ability to understand the impact of health inequality on outcomes.

This study will provide a unique landscape analysis across LMICs and HICs using standard data collection and measures of sepsis severity. It has the potential to influence future clinical practice, antimicrobial stewardship, public policy and sepsis research conduct in major urban centres globally.

## Supplementary material

10.1136/bmjopen-2025-101332 online supplemental table 1
