# Supplementary material for: Protocol for a multi-country retrospective observational paediatric sepsis epidemiological study (SENTINEL International)
Source: BMJ Open. 2025 Oct 15;15(10):e101332. doi: 10.1136/bmjopen-2025-101332 (PMC12557739; doi:10.1136/bmjopen-2025-101332)
Supplement: online supplemental table 1 [file bmjopen-15-10-s001.docx]

**Supplementary Table 1. SENTINEL International sites.**

| **Country** | **City** | **Hospital** |
| --- | --- | --- |
| Barbados | Bridgetown | Queen Elizabeth Hospital |
| Bahamas | Nassau | Princess Margaret Hospital |
| Canada | Calgary | Alberta Children's Hospital |
|  | Vancouver | BC Children's Hospital |
|  | Montreal | CHU Saint Justine |
| China | Hong Kong | Prince of Wales Hospital |
| Costa Rica | San Jose | National Children’s Hospital |
| Ethiopia | Addis Ababa | Saint Paul Hospital Millennium Medical Collage |
| Fiji | Suva | Divisional Hospital- CWM hospital |
| Ghana | Kumasi | Komfo Anokye Teaching Hospital |
| Kenya | Nairobi | Kenyatta National Hospital |
| India | Chennai | Kanchi Kamakoti CHILDS Trust Hospital |
|  | Jodhpur | AIIMS Jodhpur |
| Indonesia | Yogyakarta | Sardjito Hospital/UGM |
| Italy | Padova | University Hospital of Padova |
| Lithuania | Kaunas | LSMU Kauno Klinikos |
| Malawi | Blantyre | Kamuzu University of Health Sciences |
| Malaysia | Kuala Lumpur | Hospital Tuanku Jaafar, Hospital Tunku Azizah |
| Paraguay | Asuncion | Hospital Acosta Nu |
| Philippines | Manilla | St Luke's Medical Centre |
| Qatar | Doha | Sidra Medicine |
| Rwanda | Kigali | Kigali University teaching Hospital |
| Singapore | Singapore | KK Women’s and Children’s Hospital |
| South Korea | Seoul | Asan medical Centre |
| Spain | Bilbao | Cruces University Hospital |
| Sri Lanka | Colombo | Lady Ridgeway Hospital |
| Sweden | Stockholm | Karolinska University Hospital |
| Taiwan | Taipei | National Taiwan University Hospital |
| Tanzania | Dar es Salaam | Muhimbili National Hospital |
| Thailand | Bangkok | Siriraj Hospital |
| Turkiye | Ankara | Hacettepe University Ihsan Dogramaci Children's Hospital |
| United Kingdom | Leicester | Leicester Royal Infirmary |
| United States of America | Ohio | Nationwide Children's Hospital |
|  | Wilmington | Nemours Children’s Hospital |
