# Supplementary material for: Protocol for a multi-country retrospective observational paediatric sepsis epidemiological study (SENTINEL International)
Source: BMJ Open. 2025 Oct 15;15(10):e101332. doi: 10.1136/bmjopen-2025-101332 (PMC12557739; doi:10.1136/bmjopen-2025-101332)
Supplement: Supplementary file 2 [file bmjopen-2025-101332.reviewer_comments.pdf]

## PEER REVIEW HISTORY

BMJ Open publishes all reviews undertaken for accepted manuscripts. Reviewers are asked to complete a checklist review form and are provided with free text boxes to elaborate on their assessment. These free text comments are reproduced below.

### ARTICLE DETAILS

**Title (Provisional)**

Protocol for a multi-country retrospective observational paediatric sepsis epidemiological study (SENTINEL International)

**Authors**

Long, Elliot; Williams, Amanda; George, Shane; Hearps, Stephen; Yock-Corrales, Adriana; Pavlicich, Viviana; Krishnamurthy, Kandamaran; Seymour-Hanna, Yashica; Raman, Radhika; Choudhary, Bharat; Kusuma, Weda; Ribaya, Victoria; Mudithakumara, Nilanka; Lertamornkittior, Nichkamol; David, Antionette; Mohamed, Suleiman; Heye, Tigist Bacha; Njirammadzi-Maleta, Jenala; Appiah, John Adabie; Hitayezu, Janvier; Kunga, Mukami; Fung, Genevieve; Abdrazak, Rahimi; Chong, Shu-Ling; Wu, Jhong-Lin; Lee, Jeong-Yong; Roland, Damian; Bressan, Silvia; Loellgen, Ruth; Ballestero, Yolanda; Teksam, Ozlem; Jankauskaite, Lina; Thompson, Graham; Sabhaney, V; Shayan, Yasaman; Pifko, Elysha; Lloyd, Julia; Alansari, Khalid; Babl, Franz E

---

### VERSION 1 - REVIEW

---

| | |
|---|---|
| **Reviewer** | **1** |
| **Name** | **Mozun, Rebeca** |
| **Affiliation** | **University Children's Hospital Zürich, Department of Intersive Care and Neonatology and Children's Research Center** |
| **Date** | **24-Apr-2025** |
| **COI** | **None** |

---

This protocol describes the methods for a large international retrospective observational study to collect characteristics and outcomes of children with sepsis. The manuscript is well written, and the topic is of relevance for the community and of interest to readers. Providing additional details on the methods may help increase the clarity and overall informativeness of this protocol.

MAJOR COMMENTS

1. Abstract, page 5 & introduction, page 8: The current wording of the aims ("landscape analysis") might be unclear since this paper does not include analyses, but describes the methodology used to create the database. Please rephrase the aim of the study.

2. Abstract, page 5: Given the relevance of the work for the public, I kindly encourage the authors to also disseminate results using lay language and lay media.

3. Strengths and limitations, page 6: I suggest reflecting on the main expected barriers for case identification and data collection, to help assess the possibility of selection bias and confounding as potential limitations.

4. Introduction, page 8, and discussion, page 13: Please elaborate further on how this study is placed in the context of existing literature and current research on pediatric sepsis in the introduction and/or in the discussion (e.g. do other databases exist, how will this database differ or not from others).

5. Methods, design, page 8: I don't understand how the authors plan to conduct the mentioned diagnostic accuracy study using the database (which definitions, test / reference standards will they use?). I therefore kindly ask the authors to expand on this.

6. Methods, page 8: Please describe PPI roles and activities in more detail.

7. Methods, page 8, setting: I could not find the information on the participating sites. It would be interesting to mention these in the main text or in a figure. Also, describing how many centers were invited to participate, and how many agreed / declined and reasons why could help assess selection bias. I thus suggest including this information in the methods.

8. Methods, page 9, inclusion criteria: Please describe the rationale for the chosen criteria in the methods, as well as how they compare to those used in the development of the recent Phoenix Criteria, (Sanchez-Pinto, JAMA 2024, https://jamanetwork.com/journals/jama/fullarticle/2814296) and if discrepancies may affect comparability of findings in the discussion.

9. Methods, page 9, table 1: In my view, the items are described as aims rather than outcomes ("to create an international sepsis database", "to analyze and describe", "to apply", "to develop"). To clarify this, I suggest formulating aims and outcomes separately in different tables.

10. Methods, page 10, table 1: It is unclear to me how the feasibility of the inclusion criteria and outcomes will be measured. Please add information on this.

11. Methods, tables 1 and 2: I miss further details on the data to be collected, such as: 1) how are comorbidities defined?; 2) are other general PICU risk stratification scores like the Pediatric Index of Mortality measured?; 3) how accurately is age measured (in days / months / years)?; 4) how is ethnicity assessed and how is it categorized? (indigenous status may be insufficient for an international study); 5) how is socioeconomic status assessed? 6) is the focus of infection documented? 7) are biomarkers like PCR and PCT collected? (even if these

are not available in all sites, it would be relevant to have this information). I suggest expanding on such details, or to reflect on the lack of some information in the limitations.

12. Methods, statistical methods, page 12: The authors may consider making power calculations for the main aims/outcomes based on estimations (e.g. from Rud, The Lancet, 2020, https://www.thelancet.com/journals/lancet/article/PIIS0140-6736(19)32989-7/fulltext ) or indicating if appropriate power calculations will be included in future papers for specific outcomes using the database.

13. Methods, page 12, ethical issues: It is not clear to me if all or some centers have general consent in place and if it needs to be approved by patients/care givers, or if the authors have obtained an allowance to collect all data regardless of general consent status. I suggest clarifying this, as it has implications on the representativeness of the data.

14. Data sharing, page 13: I understand that identifiable information needs to remain in the sites. However, it should be possible to pool de-identified data from different sites to perform joint analyses. I suggest specifying how could anonymized data be accessed by collaborators and by third parties.

MINOR COMMENTS

15. Introduction, page 8, lines 10-12: add the mentioned reference.

16. Methods, design, page 8, line32: I understand several studies will be conducted using this database. I would thus suggest rephrasing “the study will follow…” to (for example) “studies conducted using this database will follow…”.

17. Figure 1: For descriptive purposes, they authors may consider adding also bullet points indicating reasons for not meeting inclusion criteria in the box for excluded patients not meeting inclusion criteria. In the box below on excluded patients, please clarify what does missing data mean in this context (i.e. which data is missing).

---

**Reviewer** 2

**Name** Tripodi, Vincenzo

**Affiliation** Azienda Ospedaliera Universitaria G Martino di Messina, Human Pathology Department

**Date** 17-Aug-2025

**COI** None

---

The authors submitted an original research protocol. The objectives and the rationale are clearly reported.

The manuscript structure and writing are well-organized and easily comprehensible.

The study design and limitations of the study are well described in the text.

This manuscript requires minor language adjustments

I suggest refining the English in the text. For example:

World Health Organization: “Organization” instead of “Organisation”;

“characterize” instead of “characterise”

“one or more fluid boluses to treat impaired perfusion, or vasoactive infusions” instead of “fluid bolus to treat impaired perfusion or vasoactive infusion”

“whichever occurs first” instead of “comes first”

“…3.4 million deaths, most of them in children younger than 5 years of age” instead of “…3.4 million deaths with most in children younger than 5 years of age”

…remains unchanged. Any reduction in sepsis mortality…” instead of “…sepsis-specific mortality remains unchanged, any reduction in sepsis mortality…”

“…based on measures of dysfunction in four organ systems after the initial 24 hours of hospitalization (Phoenix criteria)” instead of “…Phoenix criteria (based on measures of dysfunction of four organs after the initial 24 hours of hospitalisation)”

allow comparisons across diverse populations” instead of “allow for comparison between diverse populations”

“has the potential to influence future clinical practice…” instead of “impact on future clinical practice…”

Furthermore “The United Nations Millennium Development Goals (MDGs)” referred to targets set for 2015. It is therefore preferable to refer to the “Sustainable Development Goals (SDGs)”.

Additionally, I recommend including more discussion to contextualise the reported outcomes within the current literature.

Best regards.

---

## VERSION 1 - AUTHOR RESPONSE

Reviewer: 1

Dr. Rebeca Mozun, University Children's Hospital Zürich

Comments to the Author:

This protocol describes the methods for a large international retrospective observational study to collect characteristics and outcomes of children with sepsis. The manuscript is well written, and the topic is of relevance for the community and of interest to readers. Providing

additional details on the methods may help increase the clarity and overall informativeness of this protocol.

MAJOR COMMENTS

1. Abstract, page 5 & introduction, page 8: The current wording of the aims ("landscape analysis") might be unclear since this paper does not include analyses, but describes the methodology used to create the database. Please rephrase the aim of the study.

*Authors reply: we have replaced 'landscape analysis' with 'database' in the abstract and introduction.*

2. Abstract, page 5: Given the relevance of the work for the public, I kindly encourage the authors to also disseminate results using lay language and lay media.

*Authors reply: we have included 'lay media' in the dissemination plan (subject to their interest!).*

3. Strengths and limitations, page 6: I suggest reflecting on the main expected barriers for case identification and data collection, to help assess the possibility of selection bias and confounding as potential limitations.

*Authors reply: we have added the following to the Strengths and Limitations section 'The potential for variability in case identification and data collection have been mitigated using data dictionaries and standard operating procedures, site training and piloting of clinical report forms, delegation logs, and ongoing data quality monitoring in keeping with good clinical practice.'*

4. Introduction, page 8, and discussion, page 13: Please elaborate further on how this study is placed in the context of existing literature and current research on pediatric sepsis in the introduction and/or in the discussion (e.g. do other databases exist, how will this database differ or not from others).

*Authors reply: we have added 'the first' database for children with community acquired sepsis…' to the introduction, and to the Discussion: 'This is the first international*

*database…' and 'Prior global epidemiological studies have focussed on the point prevalence of sepsis in critical care populations or global estimates of disease burden(5, 27). Building on these prior efforts,…'.*

5. Methods, design, page 8: I don't understand how the authors plan to conduct the mentioned diagnostic accuracy study using the database (which definitions, test / reference standards will they use?). I therefore kindly ask the authors to expand on this.

*Authors reply: we have used this methodology in a parent database to externally validate the Phoenix sepsis criteria, and have included the following in the Methods section: '….using methodology applied to similar a bi-national (Australia and New Zealand) sepsis database' with a reference to this manuscript.*

6. Methods, page 8: Please describe PPI roles and activities in more detail.

*We have added the following to the PPI activities: 'This parent consumer and a hospital family advisory committee reviewed the study for acceptability of data collection. Their feedback will be included in publications and presentation of study results.'*

7. Methods, page 8, setting: I could not find the information on the participating sites. It would be interesting to mention these in the main text or in a figure. Also, describing how many centers were invited to participate, and how many agreed / declined and reasons why could help assess selection bias. I thus suggest including this information in the methods.

*Authors reply: We have included a list of sites as a supplementary table, from the original manuscript: 'Out of a total of 34 sites, 25 are in LMICs and 9 in HICs (see Supplemental Material)'. We included the following in Methods, setting and participants: 'Study sites were identified through known contacts. No approached sites declined participation'.*

8. Methods, page 9, inclusion criteria: Please describe the rationale for the chosen criteria in the methods, as well as how they compare to those used in the development of the recent Phoenix Criteria, (Sanchez-Pinto, JAMA 2024, https://jamanetwork.com/journals/jama/fullarticle/2814296) and if discrepancies may affect comparability of findings in the discussion.

*Authors reply: We have included the following description in the Methods, inclusion criteria: 'We chose these inclusion criteria because prior studies had indicated that an admission diagnosis of sepsis alone did not identify a substantial proportion of febrile children started on a vasoactive infusion and treated in the intensive care unit (ICU)(18). We included 'treatment for suspected sepsis' as an inclusion criterion as prior studies had identified that a substantial proportion of patients with infection requiring ICU-level care did not have an admission diagnosis of sepsis(18). This was operationalized using the administration of a fluid bolus for poor perfusion as this was the initial treatment for sepsis recommended in all guidelines and pathways at participating sites in addition to the use of intravenous antibiotics(19). The requirement for the fluid bolus to treat impaired perfusion was included as prior studies identified dehydration as the most common indication for fluid bolus administration(20). These criteria were used in a bi-national sepsis epidemiological study (SENTINEL; Australia and New Zealand) and differ from the inclusion criteria used in the derivation and validation of the Phoenix sepsis criteria (suspected infection operationalised as receipt of parenteral antimicrobials and microbiological testing within the first 24-hours of encounter)'. We have included the following explanation in the Discussion: 'The study inclusion criteria differ from those used in the derivation and validation of the Phoenix sepsis criteria, which retrospectively collected data from the pre-COVID-19 era(11). We did not use microbiological testing as an inclusion criterion because many hospitals post COVID-19 performed routine microbiological testing in children in whom sepsis was not suspected. Nevertheless, our inclusion criteria when applied to two high-income countries identified remarkably similar cohort of patients to the Phoenix derivation and validation cohort(12)'.*

9. Methods, page 9, table 1: In my view, the items are described as aims rather than outcomes ("to create an international sepsis database", "to analyze and describe", "to apply", "to develop"). To clarify this, I suggest formulating aims and outcomes separately in different tables.

*Authors reply: thank-you for pointing this out. We have re-labelled the items listed in Table 1 as aims rather than outcomes.*

10. Methods, page 10, table 1: It is unclear to me how the feasibility of the inclusion criteria and outcomes will be measured. Please add information on this.

*Authors reply: We have included the following in Methods, sample size and power calculation: 'The feasibility of inclusion criteria and outcomes for use in the design and conduct of future international interventional trials will be assessed using prevalence, severity, outcome, and treatment data from this study'.*

11. Methods, tables 1 and 2: I miss further details on the data to be collected, such as: 1) how are comorbidities defined?; 2) are other general PICU risk stratification scores like the Pediatric Index of Mortality measured?; 3) how accurately is age measured (in days / months / years)?; 4) how is ethnicity assessed and how is it categorized? (indigenous status may be insufficient for an international study); 5) how is socioeconomic status assessed? 6) is the focus of infection documented? 7) are biomarkers like PCR and PCT collected? (even if these are not available in all sites, it would be relevant to have this information). I suggest expanding on such details, or to reflect on the lack of some information in the limitations.

*Authors reply: We have included the following in Methods: 'Age was measured in months for children <2 years of age and in years for children ≥2 years of age. Indigenous status was defined using local ethnicity criteria, where applicable (e.g. Aboriginal or Torres Strait Islander in Australia; Māori or Pasifika in New Zealand). Co-morbidities were defined using the Pediatric Complex Chronic Condition system(21). We included primary site of infection and primary pathogen causing sepsis in the data collected. We did not collect inflammatory markers such as C-reactive protein or procalcitonin'. We did not collect data on socio-economic status. We have included the following in the Discussion: 'We did not collect data on inflammatory markers such as C-reactive protein or procalcitonin as they are not included in any sepsis diagnostic criteria or risk stratification criteria. We did not collect data included the Pediatric Index of Mortality-3 (PIM-3) or Pediatric Risk of Mortality-3 (PRISM-3) as these scores were developed for use in the ICU setting'.*

12. Methods, statistical methods, page 12: The authors may consider making power calculations for the main aims/outcomes based on estimations (e.g. from Rud, The Lancet, 2020, https://www.thelancet.com/journals/lancet/article/PIIS0140-6736(19)32989-7/fulltext ) or indicating if appropriate power calculations will be included in future papers for specific outcomes using the database.

*Authors reply: Thank-you for the suggestion. We have not included any power calculations as these would be based on unfounded assumptions regarding incidence, severity, and outcomes. However, we have included the following in the Methods, sample size and power calculation: 'Where applicable, appropriate power calculations will be included in future papers describing specific outcomes using the database'.*

13. Methods, page 12, ethical issues: It is not clear to me if all or some centers have general consent in place and if it needs to be approved by patients/care givers, or if the authors have obtained an allowance to collect all data regardless of general consent status. I suggest clarifying this, as it has implications on the representativeness of the data.

*Authors reply: The original submitted manuscript states: 'Each jurisdiction was consulted to determine the level of consent required. Given the observational non-interventional nature of this study, to date, no sites with IREB approval have been required to obtain consent'. As such, no study site has been required to obtain consent from parents / caregivers.*

14. Data sharing, page 13: I understand that identifiable information needs to remain in the sites. However, it should be possible to pool de-identified data from different sites to perform joint analyses. I suggest specifying how could anonymized data be accessed by collaborators and by third parties.

*Authors reply: we have included the following statement in Data Sharing: 'Aggregate data will be available upon request from the corresponding author within the constraints of ethics and regulatory requirements'.*

MINOR COMMENTS

15. Introduction, page 8, lines 10-12: add the mentioned reference.

*Authors reply: thank-you, this was our oversight! References added.*

16. Methods, design, page 8, line32: I understand several studies will be conducted using this database. I would thus suggest rephrasing "the study will follow…" to (for example) "studies conducted using this database will follow…".

*Authors reply: Thank-you for the suggestion, the text has been updated to 'Studies conducted using this database…'.*

17. Figure 1: For descriptive purposes, they authors may consider adding also bullet points indicating reasons for not meeting inclusion criteria in the box for excluded patients not meeting inclusion criteria. In the box below on excluded patients, please clarify what does missing data mean in this context (i.e. which data is missing).

*Authors reply: we have amended Figure 1 as suggested. We have included the following in Methods, patient recruitment, study procedures, and data collection: 'The reference to missing data in the figure refers to reason for exclusion'.*

Reviewer: 2
Dr. Vincenzo Tripodi, Azienda Ospedaliera Universitaria G Martino di Messina
Comments to the Author:
The authors submitted an original research protocol. The objectives and the rationale are clearly reported.
The manuscript structure and writing are well-organized and easily comprehensible.
The study design and limitations of the study are well described in the text.
This manuscript requires minor language adjustments
I suggest refining the English in the text. For example:
World Health Organization: "Organization" instead of "Organisation";
"characterize" instead of "characterise"
"one or more fluid boluses to treat impaired perfusion, or vasoactive infusions" instead of "fluid bolus to treat impaired perfusion or vasoactive infusion"
"whichever occurs first" instead of "comes first"
"…3.4 million deaths, most of them in children younger than 5 years of age" instead of "…3.4 million deaths with most in children younger than 5 years of age"
…remains unchanged. Any reduction in sepsis mortality…" instead of "…sepsis-specific mortality remains unchanged, any reduction in sepsis mortality…"
"…based on measures of dysfunction in four organ systems after the initial 24 hours of hospitalization (Phoenix criteria)" instead of "…Phoenix criteria (based on measures of dysfunction of four organs after the initial 24 hours of hospitalisation)"
allow comparisons across diverse populations" instead of "allow for comparison between

diverse populations"
"has the potential to influence future clinical practice…" instead of "impact on future clinical practice…"

*Authors reply: thank-you for the suggestions. We have used UK spelling conventions as per the BMJ Open author guidelines except for proprietary names. We have made grammatical changes as suggested.*

Furthermore "The United Nations Millennium Development Goals (MDGs)" referred to targets set for 2015. It is therefore preferable to refer to the "Sustainable Development Goals (SDGs)".

*Authors reply: thank-you for the suggestion, we have updated MDGs with SDGs.*

Additionally, I recommend including more discussion to contextualise the reported outcomes within the current literature.

*Authors reply: We have included the following in the discussion: 'Prior global epidemiological studies have focussed on the point prevalence of sepsis in critical care populations or global estimates of disease burden(5, 27). Building on these prior efforts…'.*

---

## VERSION 2 - REVIEW

---


**Reviewer** **1**

**Name** **Mozun, Rebeca**

**Affiliation** **University Children's Hospital Zürich, Department of Intersive Care and Neonatology and Children's Research Center**


**Date** **02-Oct-2025**

**COI**

---

I thank the authors for implementing all the feedback. I am pleased with the manuscript in its current form, but have a follow-up remark related the author's reply on comment 11: I would appreciate if the authors could state the lack of any socioeconomic status measurements (such as income, education level or neighborhood characteristics) as a limitation in the manuscript. Socioeconomic status is an important confounder in

epidemiological research in general and in sepsis in particular, with lower socioeconomic status being associated both with increased risk of infection and worse sepsis outcomes. Interventional randomized trials will hopefully distribute this and other confounding factors evenly between groups. However, not accounting for socioeconomic factors in statistical analyses of future observational studies may hamper understanding of health inequalities and accurate assessment of outcomes in pediatric sepsis.

---

## VERSION 2 - AUTHOR RESPONSE

Reviewer: 1
Dr. Rebeca Mozun, University Children's Hospital Zürich
Comments to the Author:
I thank the authors for implementing all the feedback. I am pleased with the manuscript in its current form, but have a follow-up remark related the author's reply on comment 11: I would appreciate if the authors could state the lack of any socioeconomic status measurements (such as income, education level or neighborhood characteristics) as a limitation in the manuscript. Socioeconomic status is an important confounder in epidemiological research in general and in sepsis in particular, with lower socioeconomic status being associated both with increased risk of infection and worse sepsis outcomes. Interventional randomized trials will hopefully distribute this and other confounding factors evenly between groups. However, not accounting for socioeconomic factors in statistical analyses of future observational studies may hamper understanding of health inequalities and accurate assessment of outcomes in pediatric sepsis.

*Authors reply: The following has been added to Limitations: 'We did not collect any socioeconomic status measurements, limiting the ability to understand the impact of health inequality on outcomes'.*

Reviewer: 1
If you have selected 'Yes' above, please provide details of any competing interests.: NA

---
